# Lung Transplantation following Emergency Pneumonectomy in a Polytraumatized 18-Year-Old

**DOI:** 10.1155/2021/5584827

**Published:** 2021-12-29

**Authors:** Pascal Gräff, Nico Bruns, Christian Kühn, Christian Krettek, Axel Haverich, Michaela Wilhelmi

**Affiliations:** ^1^Trauma Department, Hannover Medical School, Hannover, Germany; ^2^Department of Cardiac, Thoracic, Transplantation and Vascular Surgery, Hannover Medical School, Hannover, Germany

## Abstract

*Introduction*. On rare occasions, a pneumonectomy needs to be performed after a severe polytrauma. Statistically, this procedure increases the mortality rate to 50%. One option to reduce this high rate could be an organ transplantation if a matching organ can be found. However, the current literature lacks any documentation of such a case. One reason for this stems from the fact that regulations for organ transplantation are very restricted and rarely allow exceptions. In addition, the chance for survival of polytraumatized patients in need of organs in the acute phase of the therapy is often quite unsure. *Case Presentation*. In this paper, we present the case of an 18-year-old patient who was involved in a serious motorcycle accident. His injury severity score was 29, but he suffered from severe bleeding in his lung which made a pneumonectomy necessary. The procedure led to a severe deterioration of his overall condition. An ECMO therapy was initiated, which effectively only slowed the aggravation. Therefore, he was transferred to our clinic where he was stabilized temporarily. A few days later, his situation turned worse. Since he had no other chance of survival, he was scheduled for a lung transplantation and was assigned an organ. After the transplantation, he stabilized quickly and recovered almost completely. *Discussion*. In this report, we want to show that an exception to the rules for organ transplantation might make sense on rare occasions. However, to increase the chance for any success, patients must be transferred to highly specialized clinics capable of treating patients with severe conditions. This might be the only chance for those patients to survive.

## 1. Introduction

Reviewing the literature, pneumonectomy is a rare necessity in polytraumatized patients [1–3]. In a larger study covering 3000 patients with lung injuries, only 12 patients underwent resection and 4 (0.1%) pneumonectomies, respectively [4]. However, the procedure-related mortality rate within this patient population was around 50%. Some of the deaths occurred because of a refractory right heart failure as well as multiple organ failure, whereas others were not solely connected to the pneumonectomy but also to the trauma itself. Whether the pneumonectomy could have been the main reason is difficult to prove [5].

By reviewing the literature, we found no cases that chose a lung transplantation as an alternative therapy. One of the reasons certainly seems to be the fact that donor organs are not easily accessible. Furthermore, it is difficult to identify and obtain a matching organ in the special and exceptional acute posttraumatic situation in which patients will not meet standard criteria for organ donation.

## 2. Case Presentation

### 2.1. Initial Medical Condition

In this manuscript, we present the case of an 18-year-old male patient who underwent pneumonectomy and consecutive lung transplantation following a severe motorcycle accident. On admission to an external level one trauma center, he was diagnosed with severe bleeding of a pulmonary vein. His ISS was 29, which counts as a major trauma with a substantial impact on the probability of survival. During the emergency surgery, the bleeding could not be stopped, which led to a pneumonectomy of the right lung as a last chance for the surgery team to get the patient's life-threatening situation somewhat under control.

After the operation, the patient was transferred to the intensive care unit where he remained pulmonarily unstable. In expectation of a prolonged ventilation time, a surgical tracheostomy was performed early on. From the beginning, the patient lacked the ability to decarboxylate even though the ventilation was escalated, which was the reason to establish a veno-venous ECMO therapy. With this treatment, a temporary stabilization was achieved. However, soon after the respiratory conditions deteriorated, the respirator settings escalated while the ECMO was not able to solve the situation. At that time, ten days after the accident occurred, the treating physicians made the decision to transfer the patient to our clinic via a helicopter.

### 2.2. Medical Condition and Measurements after Transfer to Our Hospital

**Figure 1:**
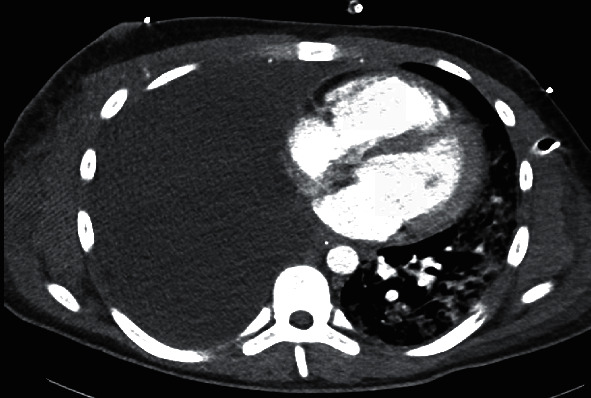
CT scan of thorax.

Our first action was to deescalate the ventilation to a lung protective setting, resulting in a quick recovery and stabilization allowing a weaning of the ECMO. However, the weaning of the ventilator and reduction of sedation put the patient under great stress resulting in tachycardia and dyspnea. Therefore, it was decided to deepen the sedation of the patient with the additional administration of a beta-blocker and later amiodarone (Figure 1).

In this CT scan, the mediastinal shift of the patient's right lung is visible on the left side of the picture, putting great pressure on the heart. The left lung shows distinct atelectasis restricting the function of the remaining lung. On the far right, the chest tube can be seen right before entering the chest cavity.

**Figure 2:**
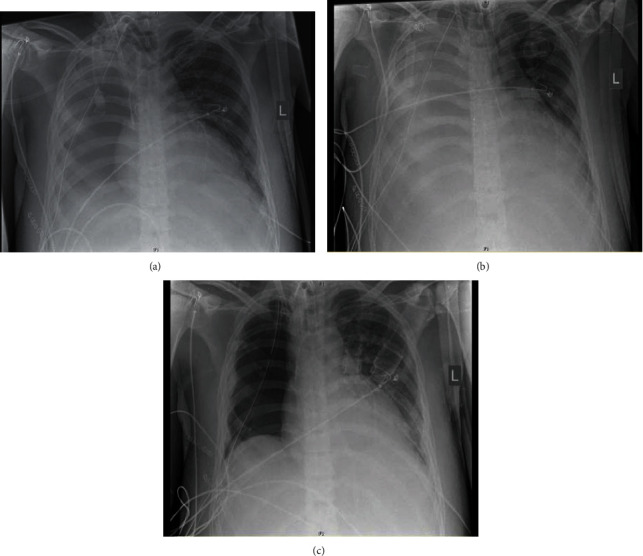
Series of chest X-rays during the time of the VATS operations.

The regularly performed X-rays showed a progressing mediastinal shift to the left, putting the heart under a lot of pressure as well as restricting the volume of the remaining left lung. The chest tube, which was still in place, was shown to be dysfunctional because of the low rate of drainage. In addition, an ongoing bleeding into the right thoracic cavity worsened the shift which made a video-assisted thoracoscopy necessary to reposition the chest tube. After the operation, the patient was stabilized for a short period of time. However, the next day, the situation was back to the preoperative status. That was why the second VATS was performed with nearly the same outcome as the first one. At that time, the patient also developed a pneumonia requiring a second veno-venous ECMO treatment. In addition, the tachycardia was not affected by the medical therapy with Amiodarone® and Metoprolol® anymore. Therefore, 3 temporarily successful cardioversions had to be performed (Figures 2(a)–2(c)).

The three chest X-rays above were taken around the time of the VATS. The left one was taken right after the first thoracoscopic operation with a centralized heart and an expanded lung.

The image in the middle was taken in the morning of the next day. The mediastinal shift appeared to be back to the way it was before. The volume of the left lung is reduced, leading to the performance of a new VATS. The third image on the right is the result of the second surgery, again without the mediastinal shift and an expanded lung. The right cavity is completely empty, which can be seen in the left part of this figure.

### 2.3. Decision for Lung Transplantation

During the following days, the pulmonary as well as the cardiac situation remained unstable. On top of that, the mediastinal shift got worse again and led to a cardiac depression which had to be treated by an ECMO conversion to a veno-veno-arterio-venous system supporting the cardiovascular system as well as the pulmonary system.

This led us to some serious consideration concerning the right treatment for the patient. How could we resolve the instability of the cardiovascular and pulmonary system? What could be a suitable and definitive plan for the weaning of the ECMO as well as the ventilator?

These problems, in our opinion, could only be addressed by a lung transplantation, which is why the patient was listed and accepted on the high urgency list.

**Figure 3:**
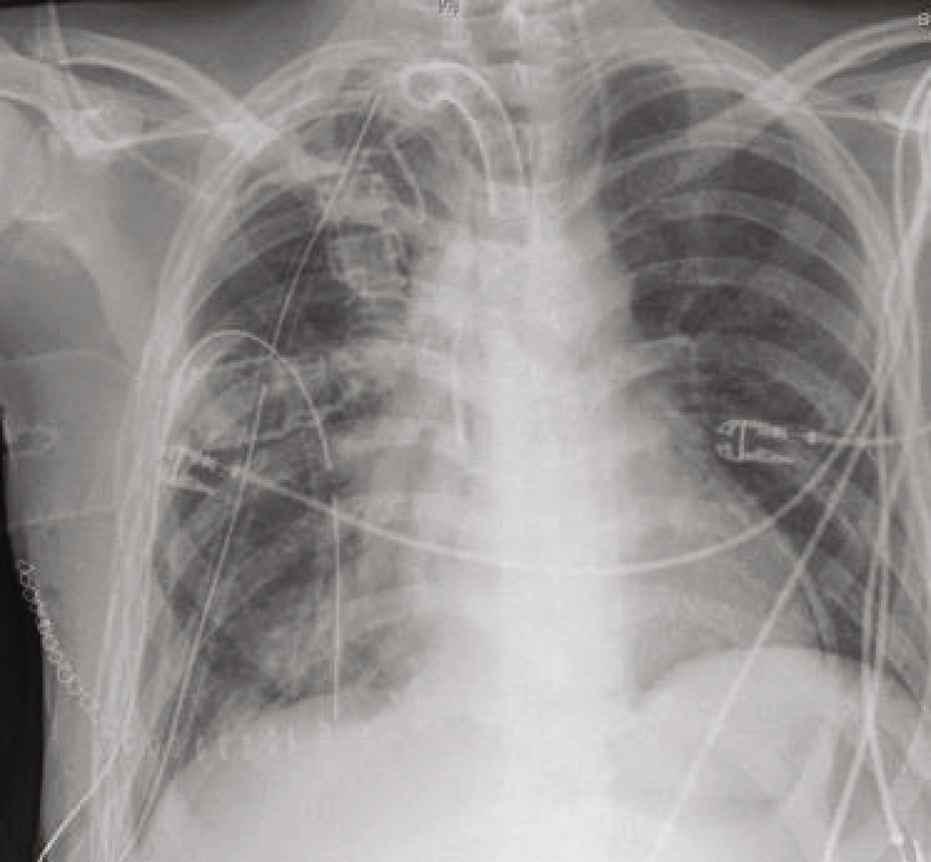
Chest X-ray after the transplantation.

During the surgery, the patient was weaned from the ECMO. After the operation, he was transferred to the specialized intensive care unit for thoracic and heart surgery where he woke up from the induced coma and was fully conscious for the first time under our treatment. Frequent thoracic X-ray examinations showed no signs of a mediastinal shift. Infection parameters like CRP and leukocytes dropped (Figure 3).

Following the transplantation, a stable situation without atelectasis is achieved; the new lung is showing on the right. The heart takes up to the half of the thorax and is in the right place without the mediastinal shift.

The heart rate normalized itself. On top of it, the tapered catecholamines as well as the antiarrhythmic medication could be reduced and subsequently dropped. During the weaning, the earlier described dyspnea did not occur again.

Two weeks after the surgery, the patient was able to breathe on his own. He was transferred to the ward where he received extensive physiotherapy to improve his mobilization ability. The transfer to a rehabilitation clinic was performed after 12 weeks as the remaining skeletal fractures had to be dealt with first.

## 3. Discussion

In conclusion, we believe that it might be appropriate in certain cases to consider an exception to the existing organ transplant regulations. In the case of our patient, it resolved almost all of his problems immediately, thus saving his life. He went through a quick pulmonary recovery and weaning without his carbon dioxide gas ever increasing to an unphysiological level again. All in all, the chosen treatment proved to be a perfectly tailored solution for this patient.

In times of limited organs available for transplantation, the strict rules for organ transplantation exist for a good reason. However, considering our case, it can be argued with some merit that exceptions to those rules might be of great importance as well. Without a transplantation, it is most likely that the patient would have died within a couple of days since his condition deteriorated every day.

The decision who might be a worthy candidate for any exceptions from the transplantation regulations is certainly a difficult one. Our patient was young and otherwise healthy with a good potential to recover and fully gets back into life. But even more important is the fact that those difficult cases belong in the right hands with experience in deciding who is most likely to recover to his or her full potential. This decision therefore requires a multiprofessional approach between highly specialized clinics.

Considering our experience with this case, we encourage every clinic treating polytraumatized patients in comparable conditions to at least consider a transplantation of an organ, especially if this might resolve the patient's problems or give him or her the only chance for survival.

## Data Availability

The data shown and used is saved in the digital archives of Hanover Medical School.
